# Characterization of *Firmiana danxiaensis* plastomes and comparative analysis of *Firmiana*: insight into its phylogeny and evolution

**DOI:** 10.1186/s12864-024-10046-2

**Published:** 2024-02-22

**Authors:** Ya-li Li, Li-yun Nie, Shuang-wen Deng, Lei Duan, Zheng-feng Wang, Joseph L.M. Charboneau, Boon-Chuan Ho, Hong-feng Chen

**Affiliations:** 1 Guangdong Provincial Key Laboratory of Applied Botany, South China Botanical Garden, Chinese Academy of Sciences, Guangzhou, 510650 China; 2 University of Chinese Academy of Sciences, Beijing, 100049 China; 3 Southern Marine Science and Engineering Guangdong Laboratory (Guangzhou), Guangzhou, 510650 China; 4 Key Laboratory of Vegetation Restoration and Management of Degraded Ecosystems, Key Laboratory of Carbon Sequestration in Terrestrial Ecosystem, South China Botanical Garden, Chinese Academy of Sciences, Guangzhou, 510650 China; 5 Department of Ecology and Evolutionary Biology, University of Michigan, Ann Arbor, MI 48109 USA; 6 Singapore Botanic Gardens, National Parks Board, 1 Cluny Road, Singapore, 259569 Republic of Singapore

**Keywords:** Plastome, Comparative genomics, *Firmiana danxiaensis*, Phylogenetic relationships, Adaptive evolution

## Abstract

**Background:**

*Firmiana danxiaensis* is a critically endangered and ecologically important tree currently only found in four locations in Danxia or Karst habitats in northern Guangdong Province, China. The specialized habitat preference makes it an ideal model species for study of adaptive evolution. Meanwhile, the phylogenetic relationships of *F. danxiaensis* in four locations under two landforms are unclear. Therefore, we sequenced its complete chloroplast (cp.) genomes and conducted comprehensive interspecific and intrageneric plastome studies.

**Results:**

The *F. danxiaensis* plastomes in four locations showed a typical quadripartite and circular structure that ranged from 160,832 to 161,206 bp in size, with 112 unique genes encoded. Comparative genomics showed that the plastomes of *F. danxiaensis* were relatively conserved with high similarity of genome organization, gene number, GC content and SSRs. While the genomes revealed higher biased codon preferences in Karst habitat than those in Danxia habitats. Eighteen and 11 divergent hotpots were identified at interspecific and intrageneric levels for species identification and further phylogenetic studies. Seven genes (clpP, accD, ccsA, ndhH, *rpl20, rpoC2, and rps4)* were under positive selection and may be related to adaptation. Phylogenetic analysis revealed that *F. danxiaensis* is sister to *F. major* and *F. simplex*. However, the interspecific relationships are not consistent with the habitat types.

**Conclusions:**

The characteristics and interspecific relationship of *F. danxiaensis* plastomes provide new insights into further integration of geographical factors, environmental factors, and genetic variations on the genomic study of *F. danxiaensis*. Together, our study will contribute to the study of species identification, population genetics, and conservation biology of *F. danxiaensis*.

**Supplementary Information:**

The online version contains supplementary material available at 10.1186/s12864-024-10046-2.

## Introduction

**Fig. 1:**
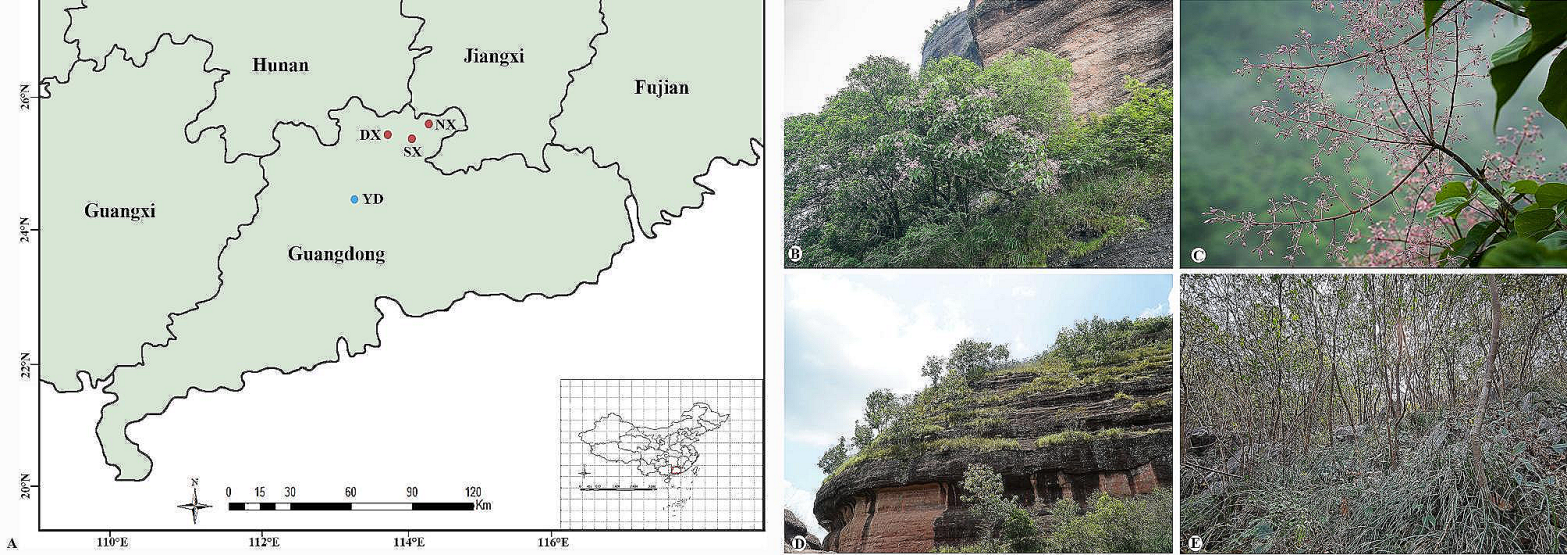
Sampling sites and habitats of Firmiana danxiaensis. SX: Shixing location. NX: Nanxiong location. DX: Danxia Mountain location. YD: Yingde location. Red circles: Danxia landform. Blue circle: Karst landform. B) Firmiana danxiaensis growing on the cliffs of Danxia habitat. C) Inflorescence of Firmiana danxiaensis. D). Danxia habitat. E) Karst habitat

*Firmiana* Marsili, a genus in the Sterculioideae subfamily of Malvaceae Juss., contains 12–18 deciduous trees or shrubs [1–4], nine of which are native to China [5–7]. Among these *Firmiana* species in China, only *F. simplex* (L.) W. Wight is not endangered [8]. Small population sizes, restricted range, and habitat specificity make them susceptible to losing genetic diversity and to a high risk of extinction. *Firmiana danxiaensis* H. H. Hsue & H. S. Kiu is a tree with significant ornamental (Fig. 1: B, C)(Fig. S1) and ecological value, which has been listed as a critically endangered (CR) species by IUCN [9]. Hitherto, *F. danxiaensis* is only found in four locations restricted to northern Guangdong Province, China: Danxia Mountain (DX) [10], Nanxiong (NX) City [11], Yingde (YD) City [12], and Shixing (SX) County [13]((Fig. 1: A). The species is naturally distributed on Danxia (DX, NX, SX) (Fig. 1: D)and Karst (YD) landforms (Fig. 1: E), both of which are considered fragile ecosystems [14]. Unique soils, such as limestone, serpentine and dolomite, are widely studied for conservation, ecology, and evolution [15]. The two landforms are edaphically isolated terrestrial habitat islands of southern China [16]. “South China Karst” and “China Danxia” were both listed as World Natural Heritage sites by UNESCO with urgent need for protection. Meanwhile, these areas were recognized as global centers of plant diversity by IUCN [17], representing priority regions for plant evolution studies. Soils derived from Danxia and limestone Karst are shallow and are washed off easily [18–20], while both are rich in mineral elements, such as calcium (Ca), magnesium (Mg) [18] and cadmium (Cd) (Table S1) (unpublished data). In addition, soils of Danxia habitats display lower concentrations of C, N, and P when compared to Karst habitats [18]. Plants in both environments are prone to drought stress due to the poor water storage capacities of Danxia and Karst soils. Harsh environmental conditions may intensify selective forces on driving evolution in plants [16].

Challenging environments may form selective genetic pressures, which leave sign of natural selection in genes involved in adaptive evolution [21]. As an essential organelle, the chloroplast (cp.) plays vital roles in plant physiology and development, such as photosynthesis, oxygen release, and amino acid and nucleotide synthesis [22]. In contrast to mitochondrial and nuclear genomes, the plastome is mainly maternally inherited in plants and has small size, slow evolutionary rate, highly conserved sequence, and many mutation events. These characteristics of the plastome, combined with modern high-throughput technology, make it an ideal model for species identification, phylogenetic reconstruction, and adaptive evolution analysis [22–25].

Earlier studies of *F. danxiaensis* mainly focus on its distribution, growth [26–29], and cultivation [30, 31]. In recent years, some studies have performed phylogenetic [32, 33] and comparative genomics analyses [34] based on *F. danxiaensis* plastomes. However, only the plastome of *F. danxiaensis* individuals from the DX location have been sequenced. Little is known about the other three locations (NX, YD, and SX). Moreover, the interspecific and intrageneric phylogenetic relationships of *F. danxiaensis* in four locations are unclear, as is whether shows signs of adaptation to Danxia and Karst habitats also remains unknown. Thus, a comparative genomics study primarily from its plastomes is urgently needed.

Here, we first sequenced *F. danxiaensis* plastomes from all four of its known locations (Table S2) and *Firmiana hainanensis*, and combined other available plastomes of *Firmiana* from GenBank to conduct comparative genomic analyses. The aims of this study were to (1) compare structures of *F. danxiaensis* plastomes in four locations; (2) detect divergent hotpots and genes under positive selection; (3) construct a phylogenetic tree both within *F. danxiaensis* and among *Firmiana* species. These results will contribute for further research on species identification, population genetics, and conservation of *F. danxiaensis.*

## Results

### Plastome features of *Firmiana danxiaensis*

**Fig. 2:**
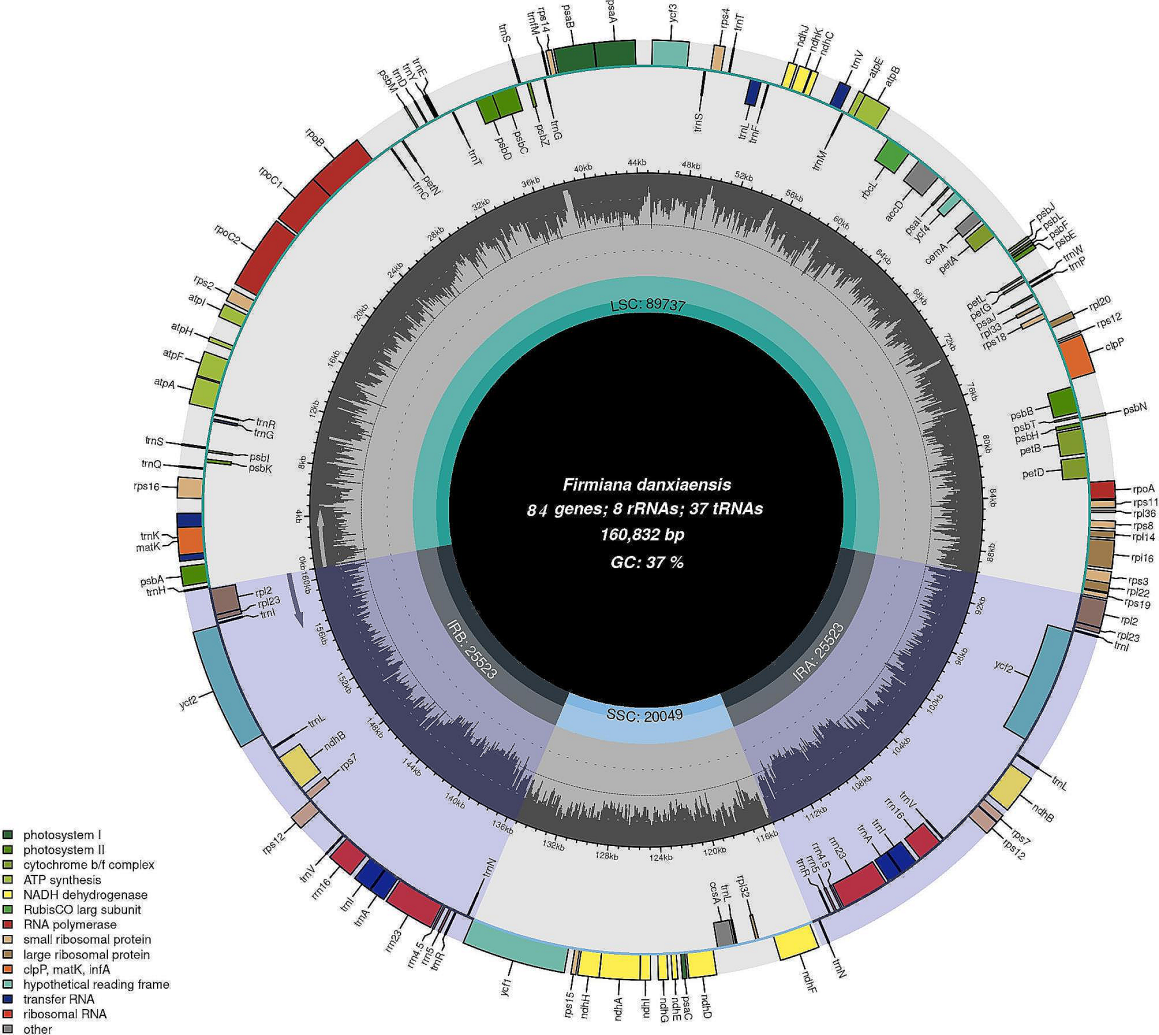
Plastome of Firmiana danxiaensis from the Shixing (SX) population. Genes are color-coded according to legend shown on the bottom left. GC content is plotted in the interior circle

The *F. danxiaensis* plastomes (GenBank accession numbers ON872508, ON872509, ON872510 and ON872511 for SX, NX, DX and YD, respectively) showed a typical quadripartite and conserved structure with almost identical length, which ranged from 160,832 to 161,206 bp (Table 1). They are composed of four parts, with the LSC region and SSC region separated by two inverted repeats, IRa and IRb (Fig. 2; Table 1). The overall GC contents of the four plastomes are the same (36.9%). While the IR regions contained higher GC content than the LSC and SSC regions, they showed few differences among the four plastomes (Table 1). The number of genes encoded by all four plastomes was identical. A total of 129 genes were encoded, of which 112 were unique, including 78 CDS genes, four rRNA genes, and 37 tRNA genes (Tables 1 and 2).

**Table 1:** Plastomes of *Firmiana danxiaensis* from four populations

Item	SX	NX	DX	YD
Plastome/ bp	160,832	160,836	161,016	161,206
LSC/ bp	89,737	89,739	89,905	90,135
SSC /bp	20,049	20,051	20,067	20,027
IR/ bp	25,523		25,522	
LSC GC content(%)	34.8	34.8	34.8	34.7
SSC GC content(%)	31.4	31.3	31.1	31.2
IR GC content(%)	42.8	42.9	43.0	43.0
GC content (%)	36.9			
Cp genes	129			
Unique CDS	78			
CDS	84			
tRNA	37			
rRNA	8			

**SX**: Shixing population. **NX**: Nanxiong population. **DX**: Danxia Mountain population. **YD**: Yingde population

**Table 2:** Gene content and functional classification of *Firmiana danxiaensis* plastome

Category	Gene Group	Gene Name
Photosynthesis	Subunits of photosystem I	*psaA, B, C, I, J*
	Subunits of photosystem II	*psbA, B, C, D, E, F, H, I,J, K, L, M, N, T, Z*
	Subunits of NADH dehydrogenase	ndhA*, B*(2), C, D, E, F, G, H, I, J, K
	Subunits of cytochrome b/f complex	petA, B*, D*, G, L, N
	Subunits of ATP synthase	atpA, B, E, F*, H, I
	Large subunit of rubisco	*rbcL*
Self-replication	Proteins of large ribosomal subunit	rpl22, 14, 16*, 2*(2), 20, 23(2), 32, 33, 36
	Proteins of small ribosomal subunit	rps16*, 11, 12*(2), 14, 15, 18, 19, 2, 3, 4, 7(2), 8
	Subunits of RNA polymerase	rpoA, B, C1*, C2
	Ribosomal RNAs	*rrn16(2), 23(2), 4.5(2), 5(2)*
	Transfer RNAs	trnA-UGC*(2), trnC-GCA, trnD-GUC,
		trnE-UUC, trnF-GAA, trnG-GCC, trnG-UCC*,
		trnH-GUG, trnI-CAU(2), trnI-GAU*(2),
		trnK-UUU*, trnL-CAA(2), trnL-UAA*,
		*trnL-UAG, trnM-CAU, trnN-GUU(2), trnP-UGG*,
		*trnQ-UUG, trnR-ACG(2), trnR-UCU, trnS-GCU*,
		*trnS-GGA, trnS-UGA, trnT-GGU, trnT-UGU*,
		trnV-GAC(2), trnV-UAC*, trnW-CCA,
		*trnY-GUA, trnfM-CAU*
Other genes	Maturase	*matK*
	Protease	clpP**
	Envelope membrane protein	*cemA*
	Acetyl-CoA carboxylase	*accD*
	c-type cytochrome synthesis gene	*ccsA*
Genes of unknown function	Conserved hypothetical chloroplast ORF	ycf1, ycf2(2), ycf3**, ycf4

Note: Gene (2), Multiple copy gene, the number of copies in parenthesis; Gene *, Gene with one intron; Gene **, Genes containing two introns

The 112 unique genes were classified into four main categories (Table 2) based on their functions. Seventeen genes were duplicated in IR regions. There were 18 genes containing introns, including 12 proteins encoding genes (rps12, rps16, rpl2, rpl16, petB, petD, ndhA, ndhB, clpP, ycf3, rpoC1, and atpF) and 6 tRNAs (trnG-UCC, trnL-UAA, trnK-UUU, trnV-UAC, trnA-UGC, and trnI-GAU), of which 16 genes have a single intron and two genes (clpP and ycf3) have two introns (Table 2). Additionally, no genes have been lost or gained in *F. danxianesis* plastomes relative to other *Firmiana* species.

### SSRs and long repeats identification

**Fig. 3:**
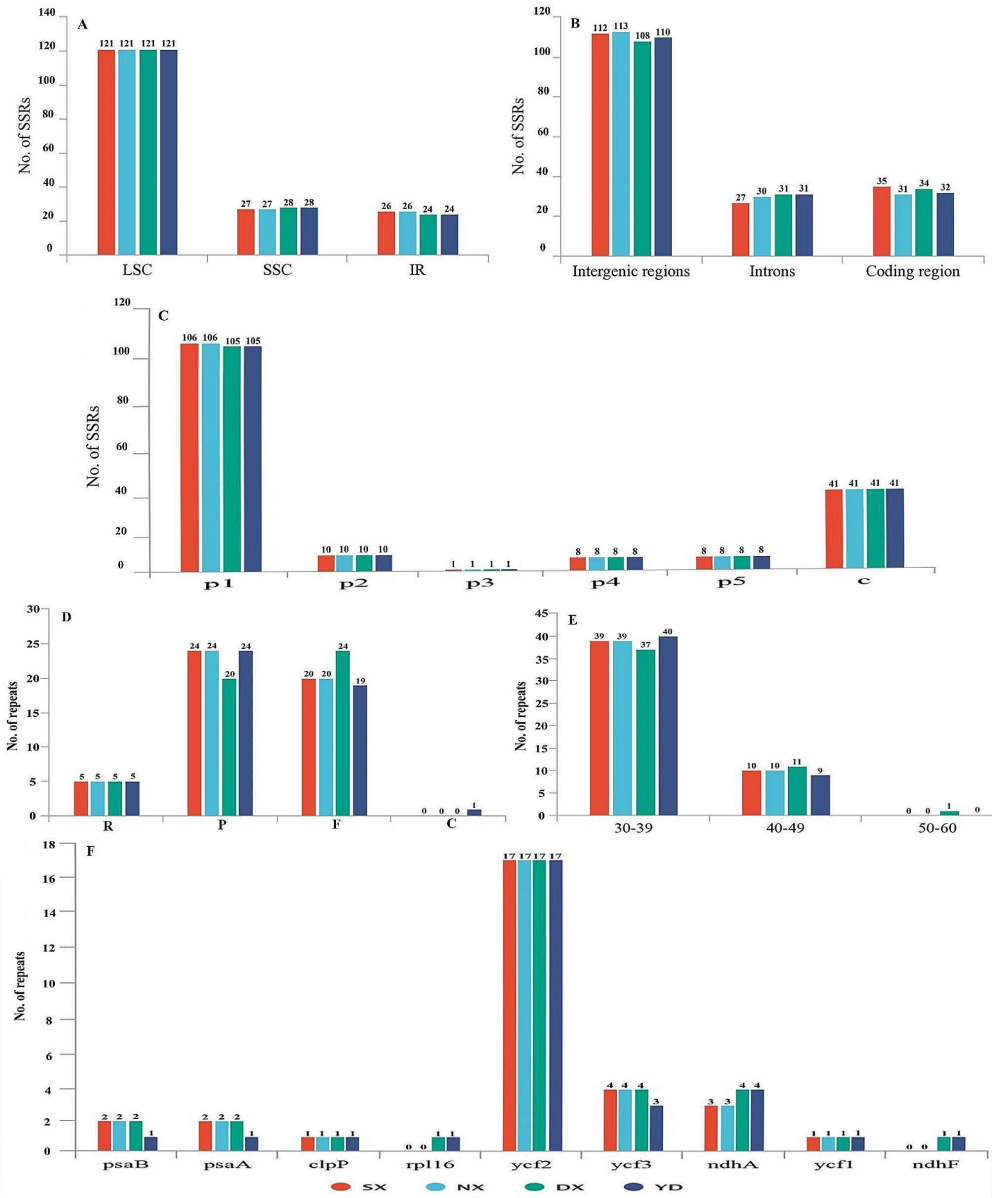
Analysis of repeated sequences of four *Firmiana danxiaensis* plastomes. **(A)** The number of SSRs distributed in different copy regions. **(B)** The number of SSRs distributed in different gene regions. **(C)** The number of six SSR types. **p1**: mononucleotide repeats. **p2**: dinucleotide repeats. **p3**: trinucleotide repeats. **p4**: tetraucleotide repeats. **p5**: pentaucleotide repeats. **c**: compound repeats. **(D)** The number of four long repeat types. **R**: reverse repeats. **P**: palindromic repeats. **F**: forward repeats. **C**: complementary repeats. **(E)** The length distribution of long repeats. **(F)** The number of long repeats located in coding genes. **SX**: Shixing population. **NX**: Nanxiong population. **DX**: Danxia Mountain population. **YD**: Yingde population

We found almost identical numbers of SSRs in four *F. danxiaensis* plastomes. SSRs were located mainly in LSC regions (69.54%), followed by SSC regions (15.85%) and IR regions (14.61%) (Fig. 3A). Additionally, 63.83% of SSRs were located in intergenic spacers, 17.24% in introns and 18.39% in coding regions (Fig. 3B). Mononucleotide repeats (p1) were the most dominant type of repeats (60.92%) among six types of SSRs (Fig. 3C). These mononucleotide repeats were mainly composed of polyadenine (poly A) and polythymine (poly T) repetitions (Table S3).

There were 49 long repeat sequences in each of the *F. danxiaensis* plastomes from four populations. Palindromic repeats (P) were the most common repeat type (46.94%), followed by forward repeats (F) (46.42%) and reverse repeats (R) (10.20%). Only the YD plastome had one complementary repeat (C) (Fig. 3D). Among the long repeats, those 30–39 bp in size were the most abundant (79.59%), followed by those 40–49 bp (20.40%). Only the DX plastome had one repeat in size of 50–60 bp (the longest was 60 bp) (Fig. 3E). Additionally, 61.22% of the long repeats were located in nine coding genes, of which ycf2 had the most repeats (Fig. 3F).

### Codon usage

For the four *F. danxiaensis* plastomes, a total of 64 types of codons encode 20 amino acids. These genomes from different populations each used an almost identical total number of codons (~ 53,656). The highest frequency of amino acid was leucine (~ 5,345 per plastome), and the lowest frequency amino acid was cysteine (~ 1,145 per plastome). In total, 33 kinds of codons had RSCU > 1 among all four plastomes (Table 3), and most of them ended with A/U. Additionally, ATG (methionine) and TGG (tryptophan) revealed no bias (RSCU = 1).

**Table 3:** Statistics of relative synonymous codon usage (RSCU) values among plastomes of *Firmiana danxiaensis* from four populations

Sample	Codon	ENC	CAI	RSCU > 1	1 < RSCU ≤ 1.2	1.2 < RSCU ≤ 1.3	RSCU > 1.3
SX	53,610	55.85	0.649	31	10	10	11
NX	53,612	55.94	0.644	30	7	11	12
DX	53,672	55.41	0.641	34	12	10	12
YD	53,735	55.13	0.648	32	13	5	14

**Note SX** Shixing population. **NX** Nanxiong population. **DX** Danxia Mountain population. **YD** Yingde population. **ENC** = Effective number of codons, **CAI** = Codon adaptation index

The highest and lowest RSCU values were recorded for AGA (1.9850) and CGC (0.4780), respectively, both encoding arginine in *F. danxiaensis* plastome of YD. Meanwhile, the genome from YD had the greatest number of high RSCU values (42.42%) (Table S4), which had highly preferred codons for arginine (AGA), aspartic acid (GAT), glutamine (CAA), and leucine (TTA).

### SNP and indels detection

**Fig. 4:**
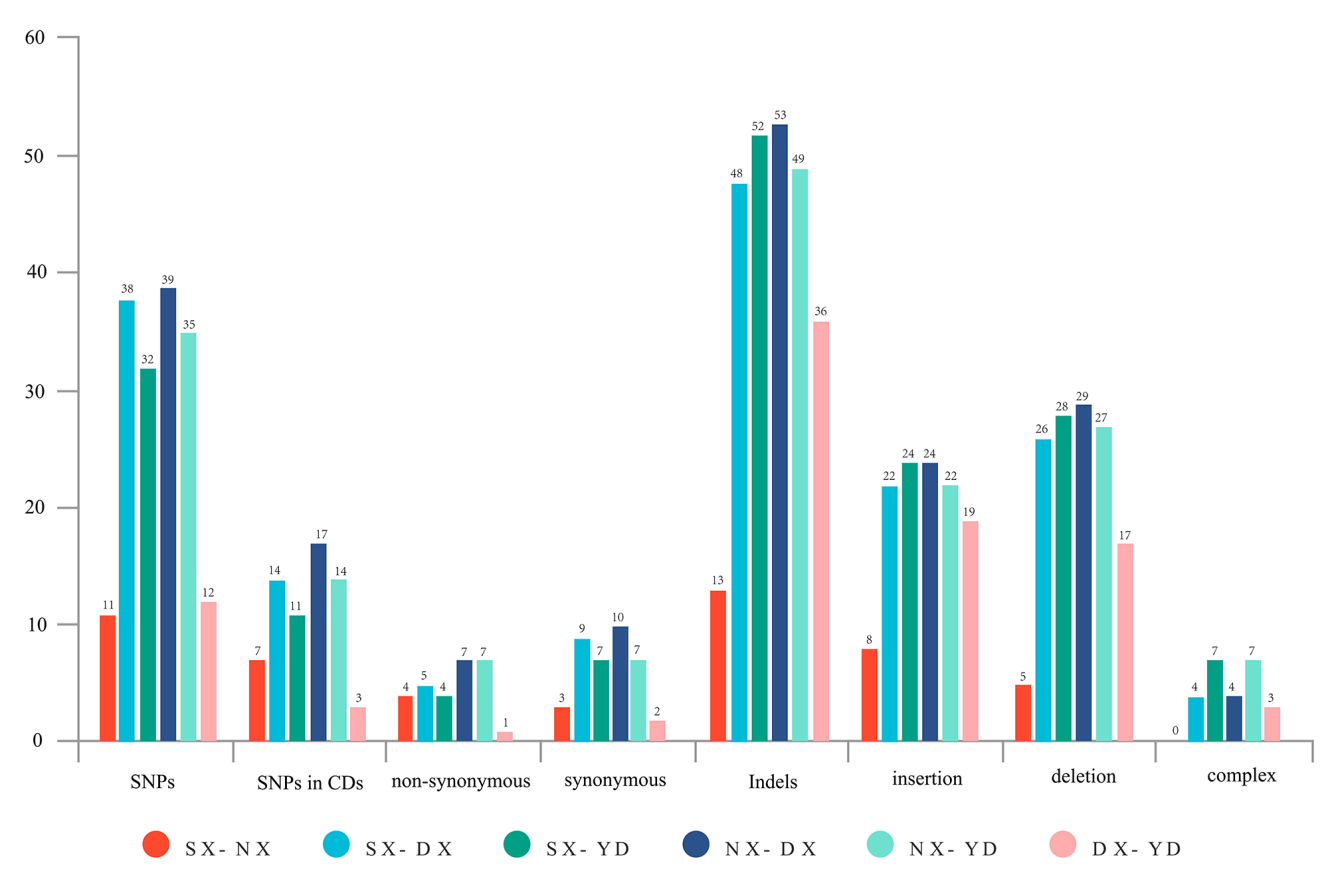
**Counts** of SNP and indels from pairwise comparisos of four *Firmiana danxiaensis* plastomes. **SX** Shixing population. **NX** Nanxiong population. **DX** Danxia Mountain population. **YD** Yingde population

SNPs and indels in four *F. danxiaensis* plastomes were compared pairwise with each other. The highest number of SNPs and indels were both found when NX plastome was compared to DX genome, and the lowest were found when SX plastome was compared to NX genome (Fig. 4). Additionally, there were 13 genes that presented variations, in which nine genes (matK, atpF, rpoC2, rpoC1, rps3, ndhE, ndhF, ycf1, and psaA) presented non-synonymous variations in at least one pair (Table S5).

### Comparative plastome analysis

**Fig. 5:**
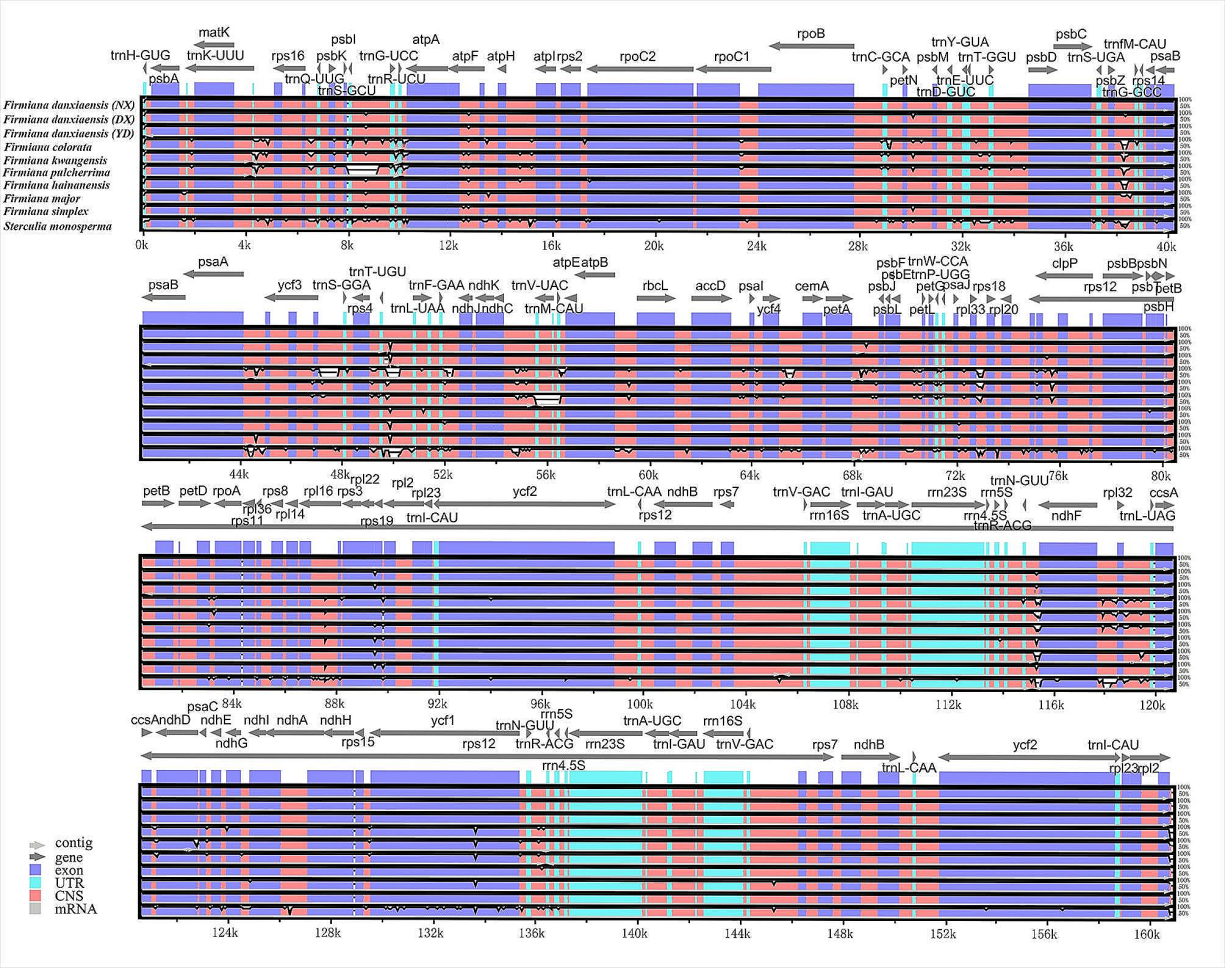
Comparisons of 11 Malvaceae plastomes. Gray arrows above the alignment indicate gene orientation. Genome regions are color-coded as exons, rRNA or tRNA (UTR), and non-coding sequences (CNS). Vertical scale indicates the percentage of identity ranging from 50 to 100%. SX: Shixing population. NX: Nanxiong population. DX: Danxia Mountain population. YD: Yingde population

Multiple alignments of 11 plastomes were compared by mVISTA with *F. danxiaensis* from SX as a reference. The results revealed a high degree of sequence similarity across these plastomes (Fig. 5), which suggested greatly conserved evolution in *Firmiana*. In general, the coding regions showed less divergence than the non-coding regions.

For four *F. danxiaensis* plastomes, rps19, psbJ, and clpP were divergent sequences in coding regions. There were six divergent sequences (trnG-UCC, atpF, trnT-UGU, trnN-GUU, trnD-GUC, trnG-GCC) in non-coding regions.

When *F. danxiaensis* plastomes were compared to *F. colorata* (Roxb.) R. Br., *F. kwangsiensis* H. H. Hsue, and *F. pulcherrima* H. H. Hsue,, rps19 and rps12 were the divergent sequences in coding regions, and trnK-UUU, trnG-GCC, and trnL-UAG were in non-protein-coding regions. Additionally, when compared to *F. hainanensis* Kosterm., *F. major* (W. W. Sm.) Hand.-Mazz., and *F. simplex*, only trnN-GUU and trnG-GCC were divergent sequences in non-protein-coding regions.

**Fig. 6:**
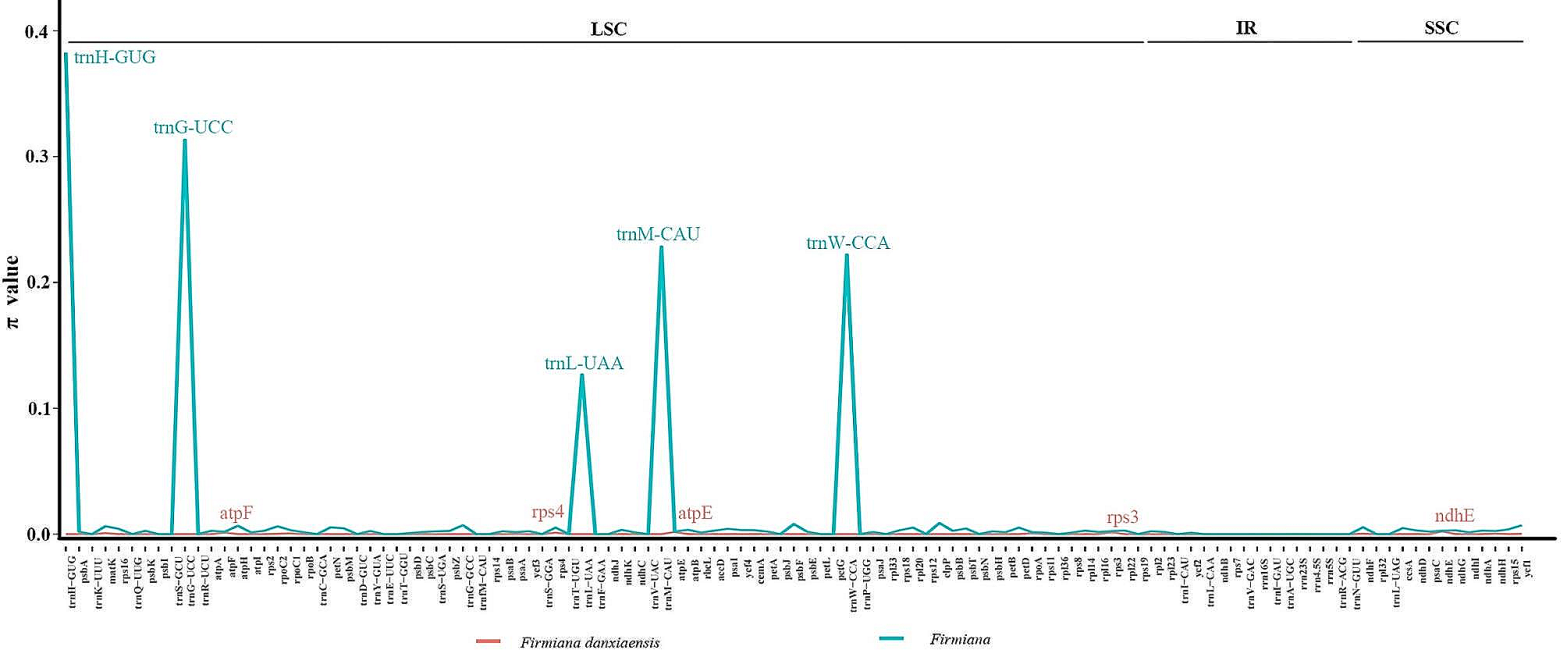
Nucleotide diversity (π) calculated from 111 loci. Pink line: π values of four Firmiana danxiaensis plastomes from four populations. Blue line: π values of 10 Firmiana plastomes. x-axis: gene name. y-axis: π value

Nucleotide diversity (π) among the four new *F. danxiaensis* plastomes calculated in 100-bp windows ranged from 0 to 0.00222, with a mean of 0.00010. Nucleotide diversity was higher in the SSC region (π = 0.00025) than the LSC region (π = 0.00001), and there was no variation within the IR region (π = 0). We detected five variable regions with relatively high π values. Four of them were in LSC region, including atpE (0.00167), rps3 (0.00153), atpF (0.00121), and rps4 (0.00111); only ndhE (0.00222) was in SSC region (Fig. 6).

Nucleotide diversity in 100-bp windows among the 10 *Firmiana* plastomes ranged from 0 to 0.38272, with a mean of 0.01332. The LSC region had higher nucleotide diversity (π = 0.01751), followed by the SSC region (π = 0.00293) and the IR (0.00031). We detected five variable regions with high π values (≥ 0.1). All of them were in LSC region, including trnH-GUG (0.38272), trnG-UCC (0.31339), trnM-CAU (0.22857), trnW-CCA (0.22222), and *trnL*-UAA (0.12698) (Fig. 6).

Overall, all 10 variable regions found within *F. danxiaensis* and among the *Firmiana* were found in the LSC or SSC regions rather than in the IR, which indicated the IR is more conserved than single-copy regions.

**Fig. 7:**
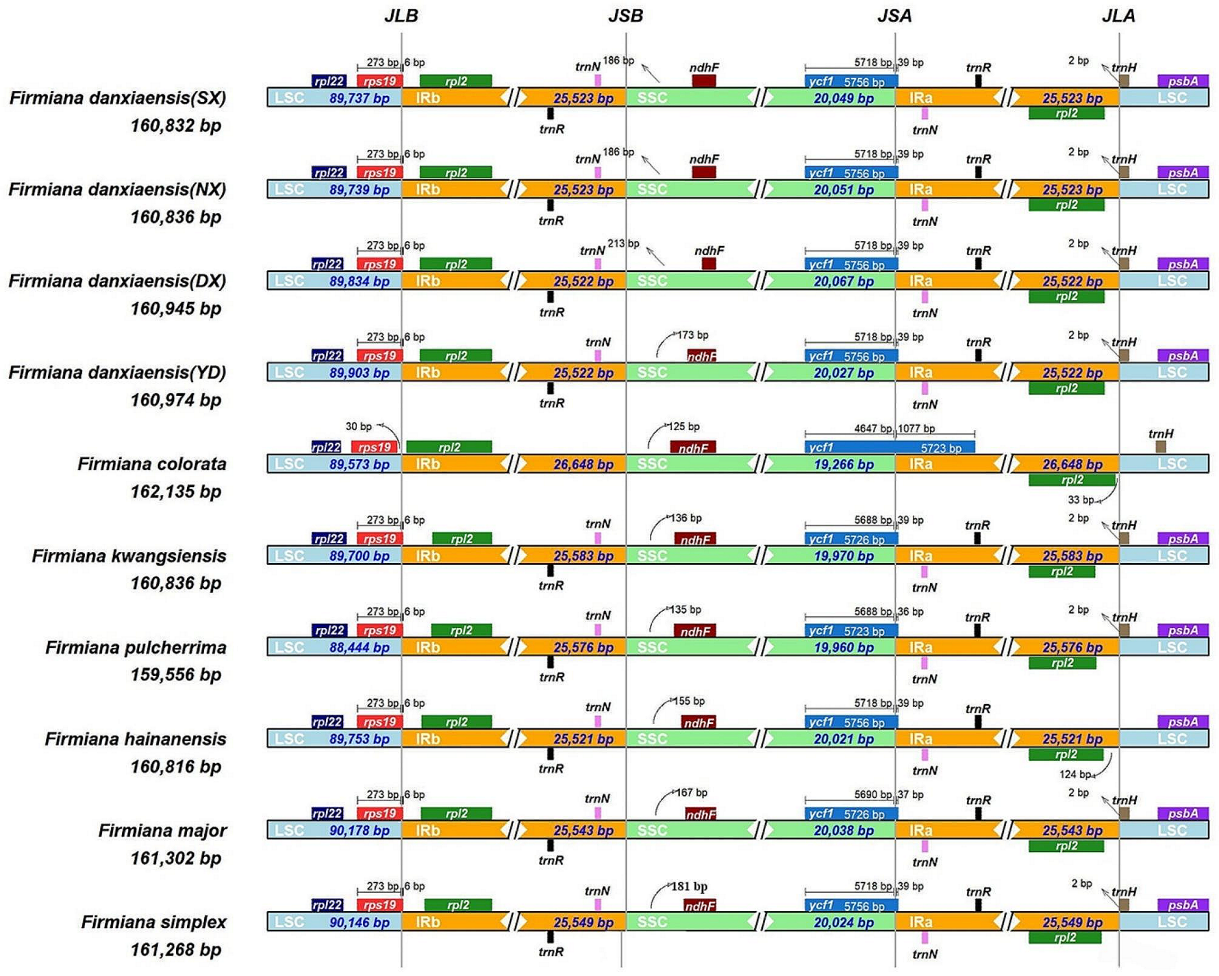
Comparison of the boundaries of the LSC, SSC, and IR regions among ten *Firmiana* plastomes. **SX**: Shixing population. **NX**: Nanxiong population. **DX**: Danxia Mountain population. **YD**: Yingde population

The IR/SC boundaries in four *F. danxiaensis* plastomes (Fig. 7) were highly consistent except for the boundary between IRb and the SSC (JSB), in which the distance between ndhF and and the end of IRb varied from 173 to 213 bp. The other three IR/SC boundaries showed there were no other expansions or contractions of the IR. In all four *F. danxiaensis* plastomes, the first 6 bp of rps19 are found in the IR at the junction between the LSC and IRb (JLB), the first 39 bp of ycf1 are in the IR at the junction between the SSC and IRa (JSA), and the last two 2 bp of trnH-GUG extend into the IR at the junction between IRa and the LSC (JLA).

The IR/SC boundaries in 10 *Firmiana* plastomes were highly consistent except in *F. colorata* (Fig. 7). At JLB, rps19 is located entirely within the LSC and its 5’ end is 30 bp away from the start of IRb. At JSA, the first 1,077 bp of ycf1 are in the IR. In *F. colorata*, there is a 33 bp gap between the start of rpl2 and the outer end of the IR as well as a 125 bp gap between the 3’ end of ndhF and the inner end of IRb (both distances are shorter than those in the other plastomes). Among the plastomes other than *F. colorata*, the distance between the JSB and ndhF varied from 135−213 bp. The other five plastomes also all had the same amount of rps19 and trnH-GUG within the IR as the *F. danxiaensis* plastomes. *F. pulcherrima* and *F. major* have 36 and 37 bp of ycf1, respectively, in the IR.

### Selective pressure analyses

When analyses of non-synonymous and synonymous substitution frequencies (Ka/Ks) were performed across eight *Firmiana* plastomes, Ka/Ks values could be obtained from 54 of 78 protein coding genes (Fig. S2). When only considering four *F. danxiaensis* plastomes, Ka/Ks values were merely available in four genes (*matK, rpoC2*, *rps4*, and *ycf1*) and they were all less than one (0.11–0.27).

**Fig. 8:**
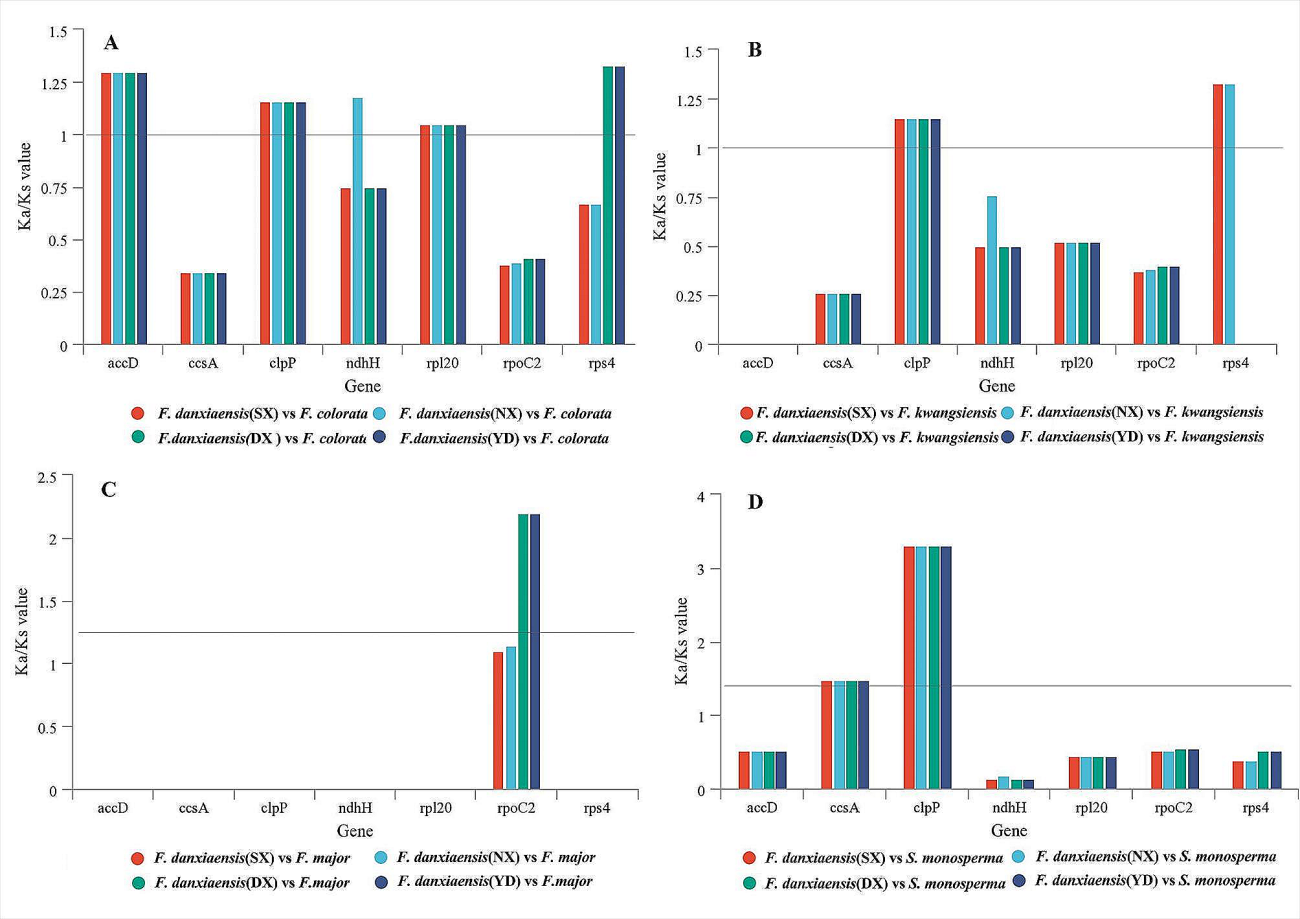
Comparison of Ka/Ks value of seven genes when Firmiana danxiaensis from four populations were pairwise compared to other four species. SX: Shixing population. NX: Nanxiong population. DX: Danxia Mountain population. YD: Yingde population

In intrageneric comparisons, the results indicated seven genes (clpP, accD, ccsA, ndhH, rpl20, rpoC2, and rps4) show signs of positive selection (Ka/Ks > 1).Among them, the Ka/Ks value of clpP gene is biggest (Ka/Ks = 3.31) when four plastome of *F. danxiaensis* from four populations were pairwise compared to *S. monosperma* (Fig. 8: D). Besides, the Ka/Ks values of four genes (accD, ccsA, clpP, and rpl20) were same when *F. danxiaensis* from four populations were pairwise compared to *F. colorata*, *F. kwangsiensis*, *F. major*, and *S. monosperma.* While, the Ka/Ks values of other 3 genes (ndhH, rps4, and rpoC2) were different (Fig. 8: A, B, C).

### Phylogenetic analyses

**Fig. 9:**
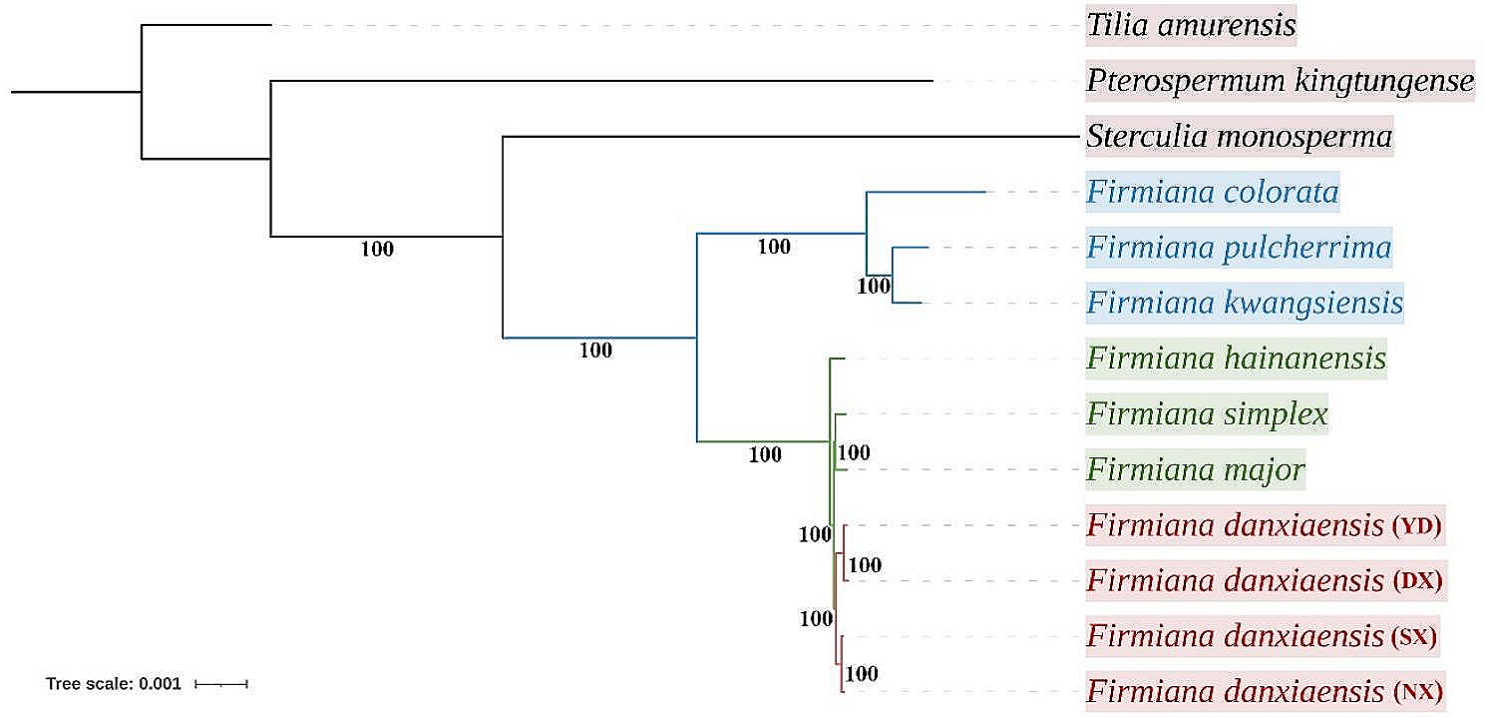
Phylogenetic relationships of *Firmiana* species. *Tilia amurensis* was set as outgroup. Phylogenetic tree was constructed by MrBayes (BI). The bootstrap values are represented at nodes

The phylogenetic trees inferred from ML and BI based on 13 whole plastomes and 78 CDS shared an identical topology, while BI showed higher support values. All branches of the BI tree (Fig. 9) had 100% Bayesian posterior probabilities. According to the topology, 10 *Firmiana* plastomes were divided into two clades, this first containing *F. colorata, F. kwangsiensis*, *and F. pulcherrima* that is sister to the second clade with the rest of species. Moreover, the *F. danxiaensis* from four populations form a clade, in which SX and NX, DX and YD show a closely genetic relationship, relatively.

## Discussion

### Conserved plastome structure and divergence hotspots analyses

The four *F. danxiaensis* plastomes exhibited a typical quadripartite structure and showed high similarities in genome sizes, structure, GC contents, and pattern of SSRs. Similar results were found in six other *Firmiana* plants [5, 34–37] (Table S6), indicating *Firmiana* plastomes are highly conserved [5].

Codon usage analysis is vital to understand the evolutionary process and selection pressure on genes [39]. For four *F. danxiaensis* plastomes, 78 genes encoded about 53,000 codons, twice as many as some species [40, 41], which may be related to codon hydrophilicity, natural selection, or gene expression rate [46]. The favored codons (RSCU > 1) tended to end in an A or U base, consistent with a previous study [47]. Additionally, the most abundant amino acids in four *F. danxiaensis* plastomes were leucine and cysteine. The same results were also reported in other species [41]. Interestingly, it was worth mentioning that RSCU values of YD differed greatly from other three locations. The highest RSCU value of any codon was found in this plastome (1.9850 for AGA encoding for arginine), accounting for the largest proportion (21.67%) of highest RSCU values among 20 amino acids as well (Table S4). Meanwhile, the YD location plastome was the only one that showed bias in the codons most commonly used for aspartic acid, glutamic acid, glutamine, and lysine. *F. danxiaensis* at the YD location is found on Karst landforms, while the other three populations are all on Danxia landforms. The soil factors concentration in two landforms are different [18], and both landforms are subjected to frequent droughts due to the poor water storage capacities of the soils. Hash environmental conditions may intensify selective forces on driving evolution in plants [16]. Thus, the higher biased codon preferences in YD plastomes might shed light on further research on adaptation evolution of *F. danxiaensis* to Karst habitat.

Regions enriched with SNPs and indels are considered hypervariable regions in the plastome [47]. In our study, there were more hypervariable regions when comparing the NX and DX plastomes than when comparing SX and NX, indicating SX and NX are more genetically closely related than the other two locations, which was accordant with the results of phylogenetic analysis (Fig. 9).

The alignments of *Firmiana* plastomes indicated that non-coding regions were more divergent than coding regions, correspondent with previous cp. genomics studies [49, 52]. For four *F. danxiaensis* plastomes, nine divergent sequences (rps19, trnG-UCC, atpF, trnT-UGU, trnN-GUU, trnD-GUC, trnG-GCC, psbJ, and clpP) could be good candidates for intraspecific identification. At the genus level, the seven divergent sequences (rps19, rps12, trnK-UUU, trnG-GCC, trnN-GUU, trnG-GCC, and trnL-UAG), some of which were also to be highly divergent in other species [38], which could be good candidates for *Firmiana* intrageneric identification.

Localized areas of high nucleotide diversity (π) can be useful molecular markers in population genetics [52]. In our study, the SC regions, especially the LSC, showed higher nucleotide diversity than the IR region., which was consistent with previous studies [41, 77]. All ten divergent regions found in interspecific and intrageneric analysis, two of which (trnL-UAA and atpE) coincide with divergent regions identified across Malvaceae [53]. Together, they could be applicable for further analysis of species identification and population genetics.

### Signature of positive selection on plastid genes

Ka/Ks values have been widely used to evaluate natural selection pressure and evolutionary rates of nucleotides in genes [54, 55], playing an important role in adaptive evolution. Our results showed that seven genes (clpP, accD, ccsA, ndhH, rpl20, rpoC2, and rps4) were subjected to positive selection, indicating that they may have contributed to habitat adaptation in *F. danxiaensis*.

Identification of positive selection of clpP were reported in other *Firmiana* or Malvaceae species (*Althaea officinalis* L., *F. major*, and *Heritiera parvifolia* Merr.) [51], and other plant families [42–47, 50] as well, indicating it may have accelerated substitution rates in many angiosperms. The clpP encodes the CLP protease, which degrades or restores damaged proteins [56, 57], and is essential for changes in plant development in response to stress [58]. Thus, positive selection in clpP gene may help explain the adaptation to harsh habitat environment; The accD gene is an essential gene required for leaf development [59], contributing to leaf longevity [59]. Actually, *F. danxiaensis* has large leaf area, accD may play roles in leaf development of *F. danxiaensis* in Danxia landform and Karst landform, which the harsh environmental conditions may intensify selective forces in plants; The ccsA gene encodes a protein required for cytochrome biogenesis which mediates the attachment of heme to c-type cytochromes [60]. The gene is also positively selected in several orchids, including epiphytic species [42, 61], of which habitat is special. Similarly, the habitats of *F. danxiaensis* are special. Thus, positive selection of ccsA gene may be related to habitat adaptation to *F. danxiaensis*; The *rpl20* gene has been evidenced to be essential for the development of cp. ribosomes in plants. Besides, all the three genes (accD, ccsA, and rpl20) also have been found to play roles in adaptation in other species [37, 41, 63].

The Ka/Ks values of ndhH, rpoC2, and rps4 genes were different in each pairwise comparisons. NADH-dehydrogenase genes groups (ndh) play vital roles in photosynthesis and they are essential in the use of light energy and the electron transfer chain to produce ATP [65, 68]. Moreover, ndh genes could allow adaptation to severe environmental stress conditions (drought, strong light or photo-oxidative stress) in the way of optimizing photosynthesis [69]; rpoC2 encodes the RNA polymerase β, which may plays role in the regulation of pollination and sex differentiation [62]; rps4 plays a role in encoding the ribosomal protein S4 [64]. *F. danxiaensis* is naturally distributed on Danxia and Karst landforms, both of which are considered fragile ecosystems [14]. Soils derived from two landforms are shallow and wash off easily [18, 19]. Such harsh environmental conditions may intensify selective forces to *F. danxiaensis* [16]. In summary, these seven positively selected genes may assist *F. danxiaensis in* adaptation to harsh environments and may serve as candidate genes for further research on the mechanisms of adaptive evolution of the species.

### Phylogenetic analyses

The phylogenetic trees (ML, BI) strongly supported intrageneric relationships of *Firmiana*, consistent with those reported in previous studies [5, 8, 70, 71], namely the two major clades within *Firmiana* recovered. Moreover, we first reported *F. danxiaensis* plastomes from the NX, SX, and YD locations, revealing the interspecific relationships of *F. danxiaensis* from all four locations in two special landforms and helped clarify the systematic relationship of *F. hainanensis* within *Firmiana*. Unexpectedly, we found the *F. danxiaensis* from YD clustered with the DX which is not consistent with the habitat types. The DX, NX and SX populations are located in Danxia landforms, while the YD population is on Karst landforms. Soils derived from Danxia and limestone Karst are significant different [18, 71]. Plants must adapt to soils for surviving and reproducing [72]. *F. danxiaensis from* NX and SX populations clustered together may be due to small geographic distance and the same type of landform. However, the geographic distance between the YD and DX populations is the largest compared to the other pairwise comparisons among four populations. The reason for the YD and DX populations cluster may be related to dispersal history of *F. danxiaensis* [48]. Meanwhile, Wang et al. [16] indicated that both habitats and climatic factors, independent of geography, can drive genomic differentiation among populations, and the effects of climatic factors can be larger than habitat type. Thus, the interspecific phylogenetic relationship and evolution of *F. danxiaensis* requires further integration of geographical distances, environmental factors, and genetic variations into genomic study.

## Conclusions

Our study supported the conserved structure of *F. danxiaensis* in four locations. Interestingly, we found the higher biased codon preference in Karst habitat than those in Danxia habitats, which sheds light on further study in adaptation of *F. danxiaensis* to Karst landform; Besides, 18 and 11 divergent hotpots were identified in interspecific and intrageneric levels for species identification and further phylogenetic studies. Moreover, seven positively selected genes (clpP, accD, ccsA, ndhH, rpl20, rpoC2, and rps4) may assist *F. danxiaensis* in adaptation to Danxia and Karst landforms. Phylogenetic analysis showed the interspecific relationships of *F. danxiaensis* are not consistent with the habitat types. Thus, further genomic study of *F. danxiaensis* with taking geographical factors, environmental factors, and genetic variations into consideration are needed. Together, our study will contribute to the study of species identification, population genetics, and conservation biology of *F. danxiaensis*.

## Materials and methods

### Sampling, DNA extraction and genome sequencing

Fresh leaves of *F. danxiaensis* were collected from four populations in Guangdong Province, China: Shixing County (SX; 114°04′ E, 25°01′ N), Nanxiong City (NX; 114°12′ E, 25°07′ N), Danxia Mountain (DX; 113°40′ E, 24°59′ N), and Yingde City (YD; 113°21′ E, 24°20′ N). Additionally, Voucher specimens of *F. danxiaensis* from four populations were deposited in the South China Botanical Garden Herbarium (Specimen number: SX: 908,669. NX: 908,661. DX: 908,667. YD: 908,663) (Table S2), which were identified by Prof. Hongfeng Chen. Leaves of *F. hainanensis* were collected from an individual cultivated in the South China Botanical Garden (SCBG), Chinese Academy of Sciences (CAS). Total genomic DNA was extracted from silica-gel dried leaves using modified CTAB methods [73]. Approximately 4 Gb of sequence data were generated for each species using the Illumina HiSeq 2000 platform with paired-end (PE) reads 150 bp in length.

### Plastome assembly and annotation

FastQC (https://www.bioinformatics.babraham.ac.uk/projects/fastqc/) was used to evaluate sequence read quality. Trimmomatic v0.39 [74] was used to remove low-quality and adapter-containing reads. The clean reads were then assembled using GetOrganelle v1.7.5 [75] with k-mers of 21, 45, 65, 85, 105, 115, 127 and max-rounds of 20. The assemblies were blasted to *F. danxiaensis* (MN720649) and *F. major* (NC_037242) genomes using Geneious R9.0.2 [76] to obtained circled genomes. CPGAVAS2 [77] was used to annotate the assembled genomes. The Chloroplot online tool [78] was used to visualize the genomes and their genes. Newly assembled genomes have been submitted to GenBank under accession numbers ON872508, ON872509, ON872510, ON872511, and ON872512 (Table S7).

### Repeat sequence analysis

Simple sequence repeats (SSRs) in the genomes were identified by MISA online software [79] with the following parameters: at least eight repeat units for mono-nucleotides, five repeat units for di-nucleotides, four repeat units for tri-nucleotides, and three repeat units for tetra-, penta-, and hexa-nucleotides. Repeat sequences (forward, reverse, complement, and palindromic repeats) were analyzed by an online REPuter software [80] with minimal repeat size of 30 bp and Hamming distance of three.

### Codon usage analysis SNP and indel detection

Relative Synonymous Codon Usage (RSCU) was analyzed by the cusp online tool (https://www.bioinformatics.nl/emboss-explorer/) with unique protein-coding genes. As reported, when RSCU ≤ 1.0, it indicates no preference for that codon for the amino acid it codes for, 1.0 < RSCU < 1.2 indicates low preference, 1.2 ≤ RSCU ≤ 1.3 indicates moderate preference, and RSCU > 1.3 indicates high preference [81]. SNPs and indels were pairwise detected among four *F. danxiaensis* plastomes using snippy [82].

### Plastome comparison

The mVISTA tool [83] was used to compare the structures of *F. danxiaensis* plastomes using the Shuffle-LAGAN mode. Four *F. danxiaensis* plastomes were compared to *F. colorata*, *F. hainanensis*, *F. kwangsiensis*, *F. major*, *F. pulcherrima*, *F. simplex*, and *Sterculia monosperma*. The IRscope online program [84] was used to compare the LSC/IRb/SSC/IRa region borders with *F. danxiaensis* plastome from SX as a reference, plus for species mentioned above in *Firmiana*. DnaSP6 software [85] was used to discover mutation hotspots with window length and step size fixed at 100 bp and 25 bp, respectively.

### Selective pressure analysis

We extracted the CDS sequences of the protein-coding genes from eight sequences (*F. danxiaensis* from four locations, *F. colorata*, *F. kwangsiensis*, *F. major*, and *S. monosperma*), which resulted in 78 CDS matrices. MAFFT [86] was used to generate CDS alignments. ALTER [87] online tool was used to convert.fasta format to.aln format. The KaKs_Calculator 2.0 [88] was used to estimate the nonsynonymous (Ka)/ synonymous (Ks) values for each gene using the γYN method. Ka/Ks < 1, Ka/Ks = 1, and Ka/Ks > 1, indicate purifying, neutral, and positive selection, respectively.

### Phylogenetic analysis

We constructed a phylogenetic tree with the newly assembled plastomes of *F. danxiaensis* and *F. hainanensis* combined eight plastomes downloaded from NCBI (Table S7). Two dataset were used to construct the phylogenetic tree, which are the CDS sequence and the whole plastomes. PhyloSuite [89] software was used to create Maximum Likelihood (ML) tree and Bayesian (BI) trees. ML analysis was performed using IQ-TREE [90] with an edge-linked partition model (K81u + I + G4 + F) and 5000 standard non-parametric bootstrap replicates. BI analysis was performed using MrBayes3.2.6 [91] with default model, two independent parallel chains and 5,000,000 generations and sampling once every 1,000 generations. The first 25% of trees from all runs were discarded as burn-in. *Tilia amurensis* Rupr. was used as outgroup. Finally, ITOL [92] software was used to visualize and refine the tree.

### Electronic supplementary material

Below is the link to the electronic supplementary material.

Supplementary Material 1

Supplementary Material 2

Supplementary Material 3

Supplementary Material 4

## Data Availability

The sequence data of *Firmiana danxiaensis* and *Firmianan hainanensisi* plastomes involved in this study have been deposited in GenBank with accession numbers ON872508 (SX), ON872509 (NX), ON872510 (DX), ON872511 (YD), and ON872512. All relevant data can be found within the manuscript and its supporting materials.

## Abbreviations

LSC: Large single-copy region
SSC: Small single-copy region
IR: Inverted Repeat
CDS: Coding sequence
RSCU: Relative synonymous codon usage
SSR: Simple sequence repeats
π: Nucleotide diversity
ML: Maximum likelihood
BI: Bayesian inference
NCBI: National Center for Biotechnology Information
SX: Shixing location
NX: Nanxiong location
DX: Danxia Mountain location
YD: Yingde location
