# Supplementary material for: Characterization of Firmiana danxiaensis plastomes and comparative analysis of Firmiana: insight into its phylogeny and evolution
Source: BMC Genomics. 2024 Feb 22;25:203. doi: 10.1186/s12864-024-10046-2 (PMC10885454; doi:10.1186/s12864-024-10046-2)
Supplement: Supplementary file 1 — Supplementary Material 1 [file 12864_2024_10046_MOESM1_ESM.docx]

**Figure S1 lengends**

**Fig. S1** Flower and fruit Sturcture of *Firmiana danxiaensis***. A**: Female flower. **B**: Male flower. **C**: Young fruits. **D**: Mature fruits.

**Figure S2 lengends**

**Fig. S2** Line chart of KaKs value range of 54 protein coding genes in eight *Firmiana* chloroplast genomes. **Red circle**: The lowest Ka/Ks value of each protein coding genes in eight *Firmiana* chloroplast genomes. **Blue circle**: The highest Ka/Ks value of each protein coding genes in eight *Firmiana* chloroplast genomes.
