# Supplementary figures and images for: Characterization of Firmiana danxiaensis plastomes and comparative analysis of Firmiana: insight into its phylogeny and evolution
Source: BMC Genomics. 2024 Feb 22;25:203. doi: 10.1186/s12864-024-10046-2 (PMC10885454; doi:10.1186/s12864-024-10046-2)

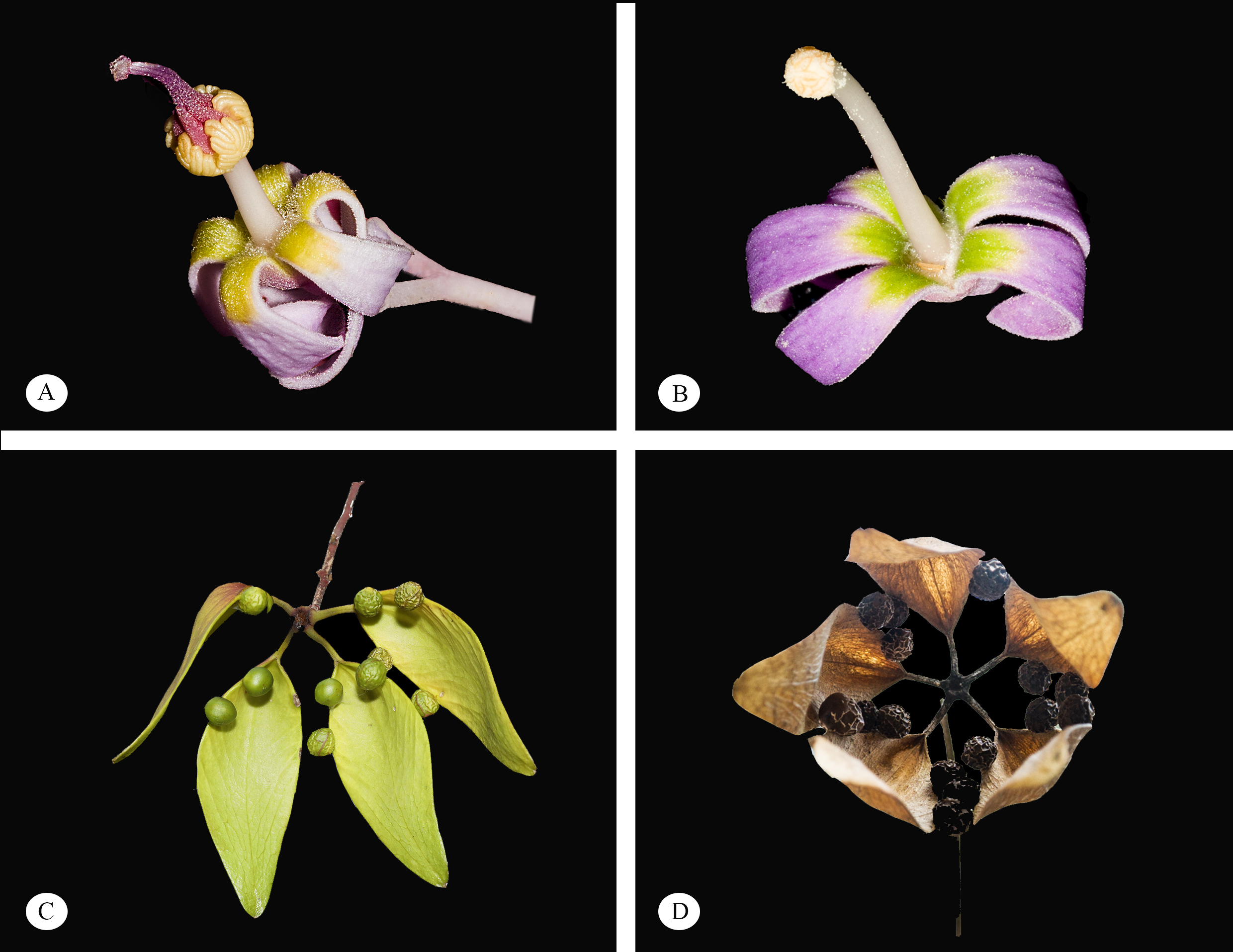

Supplement: Supplementary file 2 — Supplementary Material 2 [file 12864_2024_10046_MOESM2_ESM.jpg]

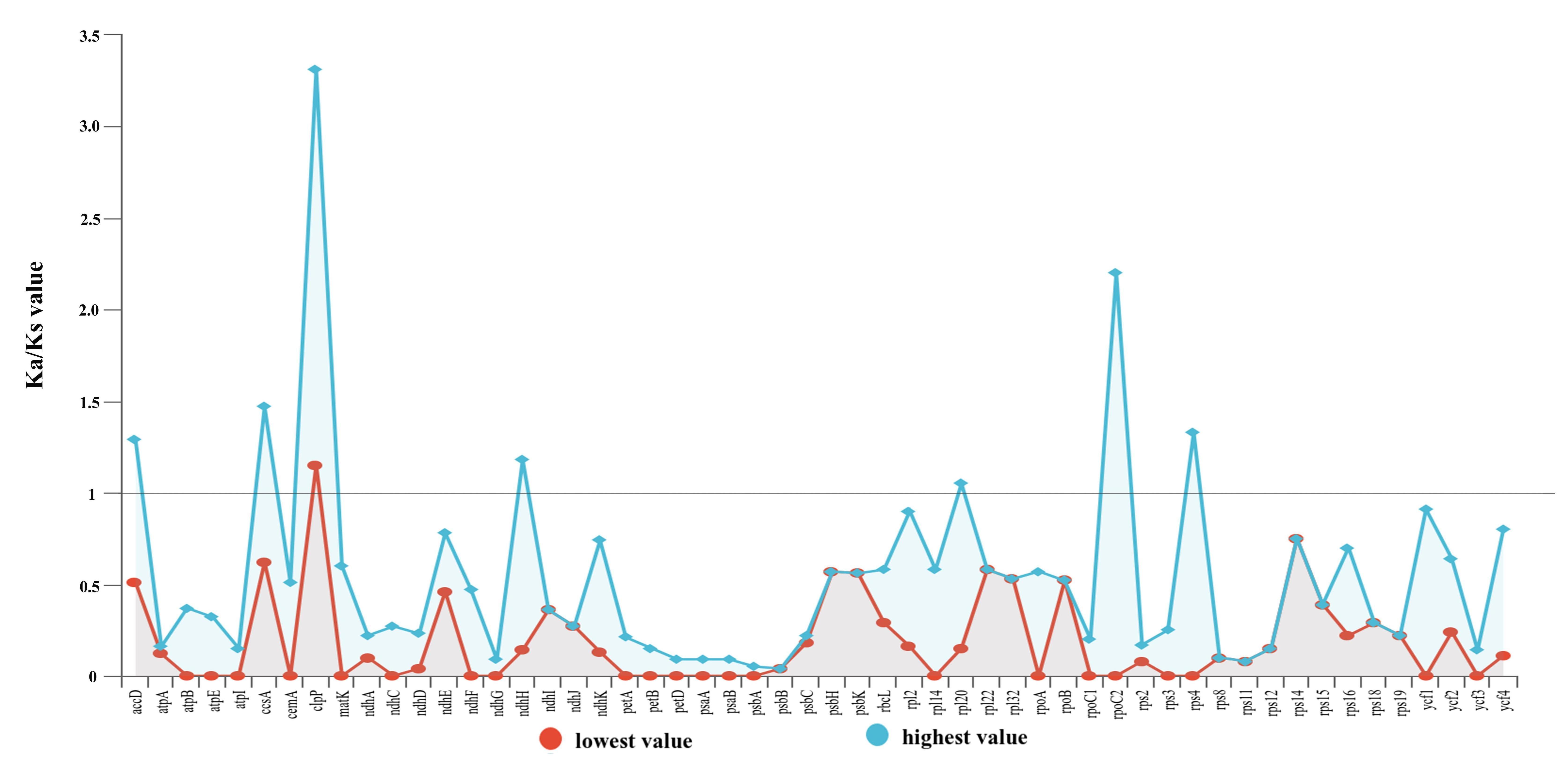

Supplement: Supplementary file 3 — Supplementary Material 3 [file 12864_2024_10046_MOESM3_ESM.jpg]
